# Anhedonia difference between major depressive disorder and bipolar disorder II

**DOI:** 10.1186/s12888-021-03548-w

**Published:** 2021-10-27

**Authors:** Xinyu Fang, Dandan Wang, Wei Tang, Hongyang Liu, Xiangrong Zhang, Chen Zhang

**Affiliations:** 1 Department of Geriatric Psychiatry, Affiliated Nanjing Brain Hospital, Nanjing Medical University, Nanjing, People’s Republic of China; 2 Shanghai Mental Health Center, Shanghai Jiao Tong University School of Medicine, Shanghai, People’s Republic of China; 3 The Affiliated Kangning Hospital of Wenzhou Medical University, Wenzhou, People’s Republic of China

**Keywords:** Major depressive disorder, Bipolar disorder, Anhedonia Snaith-Hamilton pleasure scale, Drug-free

## Abstract

**Objective:**

This study aims to explore the difference in anhedonia between Major Depressive Disorder (MDD) and Bipolar Disorder II (BD-II), and attempt to distinguish the two diseases through Snaith-Hamilton Pleasure Scale (SHAPS).

**Methods:**

A total of 164 drug-free depressive patients (98 MDD patients, 66 BD-II patients) completed the investigation. 17-item Hamilton Depression Scale (HAMD-17) and Hamilton Anxiety Scale (HAMA) and SHAPS were assessed in all participants.

**Results:**

Our results showed that BD-II patients had higher SHAPS scores than MDD patients. The stepwise logistic regression analysis further revealed that SHAPS score, drinking habit, and extroversion as influencing factors for the identification of BD-II. The ROC curve analysis indicated that SHAPS could differentiate BD-II from MDD patients (AUC = 0.655, *P* = 0.001, 95% CI = 0.568 to 0.742), with the best screening cutoff at 26, and the corresponding sensitivity and specificity was 0.788 and 0.520, respectively.

**Conclusion:**

Our results suggest that BD-II patients had more severe anhedonia compared to MDD patients, and the difference in anhedonia may help clinicians preliminary identify BD patients from MDD patients. The preliminary findings are worthly of further exploration.

**Supplementary Information:**

The online version contains supplementary material available at 10.1186/s12888-021-03548-w.

## Introduction

Major depressive disorder (MDD) and bipolar disorder (BD) are both prevalent and debilitating mood disorders [1], which cause a large burden of disease to the family and society across the world [2, 3]. A recent national survey of mental disorders conducted in China demonstrated the lifetime prevalence of MDD and BD is 3.4 and 0.6% respectively [4]. BD is characterized by recurrent episodes of depression and elevation of mood (mania and/or hypomania). Since the depressive symptoms are both common in patients with MDD and BD, and the diagnosis of MDD and BD currently relies on evaluation of symptoms by clinicians [5]. Thus, leads to a high rate of misdiagnosis between these two diseases in clinical practice, especially for BD-II patients, whose hypomanic symptoms in BD have not been recognized, or are yet to appear [6]. A national survey reported that the misdiagnosis rate for BD reaches as high as 69%, and only 20% of BD patients with a current depressive episode were correctly diagnosis within the first year of treatment [7]. What’s worse, over one-third of BD patients were still not given a confirmed diagnosis after 10 years of seeking treatment [8]. In clinical practice, the differential diagnosis of depressive episodes of MDD and BD is of great importance, since the drug treatment strategies for relieving depressive symptoms in these two disorders are distinctly different [9]. The misdiagnosis of BD along with nonideal treatments further results in worsen outcomes, including switching to manic, prolonging illness duration, increasing risk of recurrence and suicide [6, 10]. Therefore, identification of reliable tools and biomarkers for accurate differential diagnosis of MDD and BD is of enormous clinical importance.

Up to now, substantial existing research aimed to find biomarkers to discriminate BD from MDD, and some achievement has indeed been made. Since studies of twins and adoptees suggest a genetic predisposition to MDD and BD, some studies found that MDD and BD had different genetic backgrounds, which provide information for differential diagnosis [11, 12]. Our co-author Chen Zhang has also done a lot of research work on it and revealed some biochemical signatures that could be used for distinguishing BD from MDD, including inflammatory cytokines, brain-derived neurotrophic factor (BDNF), B2RAN2, and ENG proteins [13–15]. In addition, distinct gut microbes, brain structure and functions evaluated by electroencephalogram (EEG) or magnetic resonance imaging (MRI) were all used to classify BD and MDD patients [6, 9, 16]. However, these findings were poorly repeated by other studies and are still many years away from use in hospitals and clinics.

In clinical practice, it is more urgent to have a convenient tool to assist clinicians in early differential diagnosis of BD and MDD. Hence, some symptoms rating scales were used. The Mood Disorder Questionnaire (MDQ) has been used effectively in the past, which can screen for a lifetime history of manic symptoms in patients with depressive episode [17]. However, the MDQ may be insensitive in the detection of BD when previous manic or hypomanic symptoms have not been appeared or well recalled. As recent evidence showed a significant difference in somatic symptoms between MDD patients with and without persistent depressive episode [18], other researchers further revealed that MDD patients had more somatic symptoms evaluated by Patients Health questionnaire-15 (PHQ-15) compared to BD-II patients [19]. As we know, anhedonia, a lack of pleasure in response to rewarding stimuli, is a core feature of depression. Ample studies have consistently demonstrated structural and functional aberrance in reward system across patients with BD and MDD [20–22]. These findings suggest that the neural mechanism underlying the anhedonia in BD and MDD might be distinct. However, whether the severity of the anhedonia is different between BD and MDD remains unclear.

In the present study, we used a dedicated tool, the Snaith-Hamilton Pleasure Scale (SHAPS), to evaluate anhedonic symptoms in drug-free patients with MDD and BD-II. We aimed to compare the demographic and clinical differences, including the severity of anhedonia between those two patient groups, and to verify whether SHAPS could assist clinicians to discriminate BD-II from MDD initially.

## Materials and methods

### Participants

Ninety-eight MDD patients and 66 BD patients in depressive episode were recruited consecutively from Wenzhou Kangning Hospital, Wenzhou Medical University. Each patient was interviewed by two experienced psychiatrists using the Structured Clinical Interview for DSM-IV-TR-Patient Edition (SCID-P) and was finally diagnosed with BD or MDD according to DSM-IV. The patients were included only when the diagnosis was consistent between the two psychiatrists. The inclusion criteria are as follows: (1) aged 18–50 years old; (2) total score on the Hamilton Rating Scale for Depression-17 (HAMD-17) ≥ 17; (3) had a junior high school education or above, understanding and reading fluently in Chinese; (4) did not take any antidepressants during the period of 3 months before enrolment. Exclusion criteria included: (1) comorbid other Axis I psychiatric disorders including those with anxiety disorder, schizophrenia, schizoaffective disorder, or another psychotic disorder; (2) organic brain disease; (3) and those who were pregnant or breastfeeding. All participants provided written informed consent to participate in this study, which was approved by the local Medical Ethics Committee of the Wenzhou Kangning Hospital, and was performed in strict accordance with the Declaration of Helsinki and other relevant national and international regulations.

### Date collection

All participants were interviewed face-to-face. Basic demographic information (age, gender, marital and educational status) and details of the course of the patient’s illness, such as age of onset and total disease courses, were obtained by interviewing patients and caregivers, supplemented by their existing medical records. The severity of depressive or anxiety symptoms in all participants was assessed using 17-item Hamilton Depression Scale (HAMD-17) and Hamilton Anxiety Scale (HAMA), the most common tools used by clinician rating of depressive and anxiety symptoms severity. The clinical assessment was conducted by two experienced psychiatrists who were well trained for this project, and repeated assessments for the HAMD-17 or HAMA total score maintained an interrater correlation coefficient greater than 0.8. We used the Snaith-Hamilton Pleasure Scale (SHAPS), Chinese versions, to evaluate the anhedonic symptoms in depressive patients. It is a 14-item self-report questionnaire, which rated on a 4-point Likert scale from definitely agree to definitely disagree. The total score ranges from 14 to 56 for the Chinese version of the SHAPS. The higher total SHAPS scores indicate a higher level of anhedonia. It works well in anhedonic assessment and exhibited good reliability and validity with Chinese population [23], and has been widely used to assess anhedonic symptoms in depressed patients in China [24, 25].

### Statistical analysis

Data were analyzed using the Statistical Package for the Social Sciences (SPSS), version 23.0. The statistically significant level was set in alpha ≤0.05 with two-tailed. Clinical and demographic data between MDD and BD-II patients were analyzed by Student’s *t*-test for the continuous variables and the chi-squared test for categorical variables. The G*Power 3.1.9.2 program (https://www.softpedia.com/get/Science-CAD/ G-Power.shtml) was used to run a power calculation and determine the effect size of the continuous variables. The stepwise logistic regression analysis was performed to explore influence factors for the identification of BD-II patients. Finally, the receiver operating characteristic (ROC) curve was used to determine whether the patients with BD and MDD could be differentiated and to ascertain the sensitivity (SEN) and specificity (SPE) at various cutoffs. The best cutoff maximizing the sums of the SEN and SPE were calculated for the SHAPS to discriminate between MDD and BD. The criterion validity of the SHAPS was estimated using the SEN, SPE, false positive rate (FPR), false negative rate (FNR), Youden index and the area under the curve (AUC).

## Results

### Demographic and clinical variables in MDD and BD-II patients

A total of 164 participants were all outpatients, the demographic and clinical characteristics of drug-free patients with MDD and BD-II are presented in Table 1. We found no significant differences between-group differences with regards age, sex, height, weight, education levels, and marital status (All *P* > 0.05). BD-II patients tend to be more extroverted (X^2^ = 8.283, *P* = 0.016), and have higher rates for drinking (X^2^ = 6.408, *P* = 0.011) and smoking (X^2^ = 6.051, *P* = 0.014) compared to MDD patients. There were also no significant differences in age of onset, disease courses, and family history of mental illness between BD-II and MDD patients (All *P* > 0.05). For clinical symptoms, our results showed that BD-II patients had more severe anhedonic symptoms than MDD patients (t = 3.522, *P* = 0.001). However, HAMD-17 and HAMA scores show no significant differences between those two patient groups (Both P > 0.05). The power calculation showed that the statistical power for the SHAPS reached 96.78%, which indicated a high statistical power of our sample size to detect the difference of SHAPS score between the BD-II and MDD patients. Our stepwise logistic regression analysis further revealed that SHAPS score (β = 0.108, Wald X^2^ = 12.031, *P* = 0.001), drinking habit (β = 1.214, Wald X^2^ = 8.422, *P* = 0.004) and extroversion (β = − 0.416, Wald X^2^ = 5.104, *P* = 0.024) as important influencing factors for the identification of BD-II.

**Table 1:** Comparisons between drug-naïve MDD patients and BD-II patients

	MDD (*N* = 98)	BD-II (*N* = 66)	t/X^2^	*P*
Age (year)	30.37 ± 7.92	29.94 ± 8.32	0.333	0.740
Sex			1.167	0.280
Male	32	27		
Female	66	39		
Height (cm)	167.03 ± 7.10	166.68 ± 7.28	0.306	0.760
Weight (kg)	58.46 ± 10.49	60.41 ± 14.11	0.958	0.340
Character			8.283	0.016
Extrovert	33	37		
Ambivert	14	5		
Introvert	51	24		
Marital Status			0.702	0.704
Unmarried	53	32		
Married	40	31		
Divorced/Widowed	4	2		
Drinking			6.408	0.011
No	82	44		
Yes	16	22		
Education (year)			2.842	0.092
≤12	36	16		
> 12	62	50		
Smoking			6.051	0.014
No	85	47		
Yes	13	19		
Age of onset (year)	29.54 ± 8.36	28.71 ± 8.70	0.613	0.541
Total disease course (month)	11.07 ± 15.70	18.38 ± 30.62	1.787	0.077
Family history			0.338	0.561
Yes	20	16		
No	78	50		
HAMD-17	23.62 ± 4.48	22.74 ± 3.58	1.393	0.166
HAMA	19.11 ± 3.36	18.29 ± 3.15	1.579	0.116
SHAPS	24.58 ± 5.54	27.96 ± 6.66	3.522	0.001

*Abbreviations*: *MDD* major depressive disorder, *BD* bipolar disorder, *BMI* body mass index, *HAMD* Hamilton Rating Scale for Depression, *HAMA* Hamilton Rating Scale for Anxiety, *SHAPS* Snaith-Hamilton Pleasure Scale

Data were presented in Mean ± SD or N

### ROC curve analysis of SHAPS between MD and BD-II patients

**Fig. 1:**
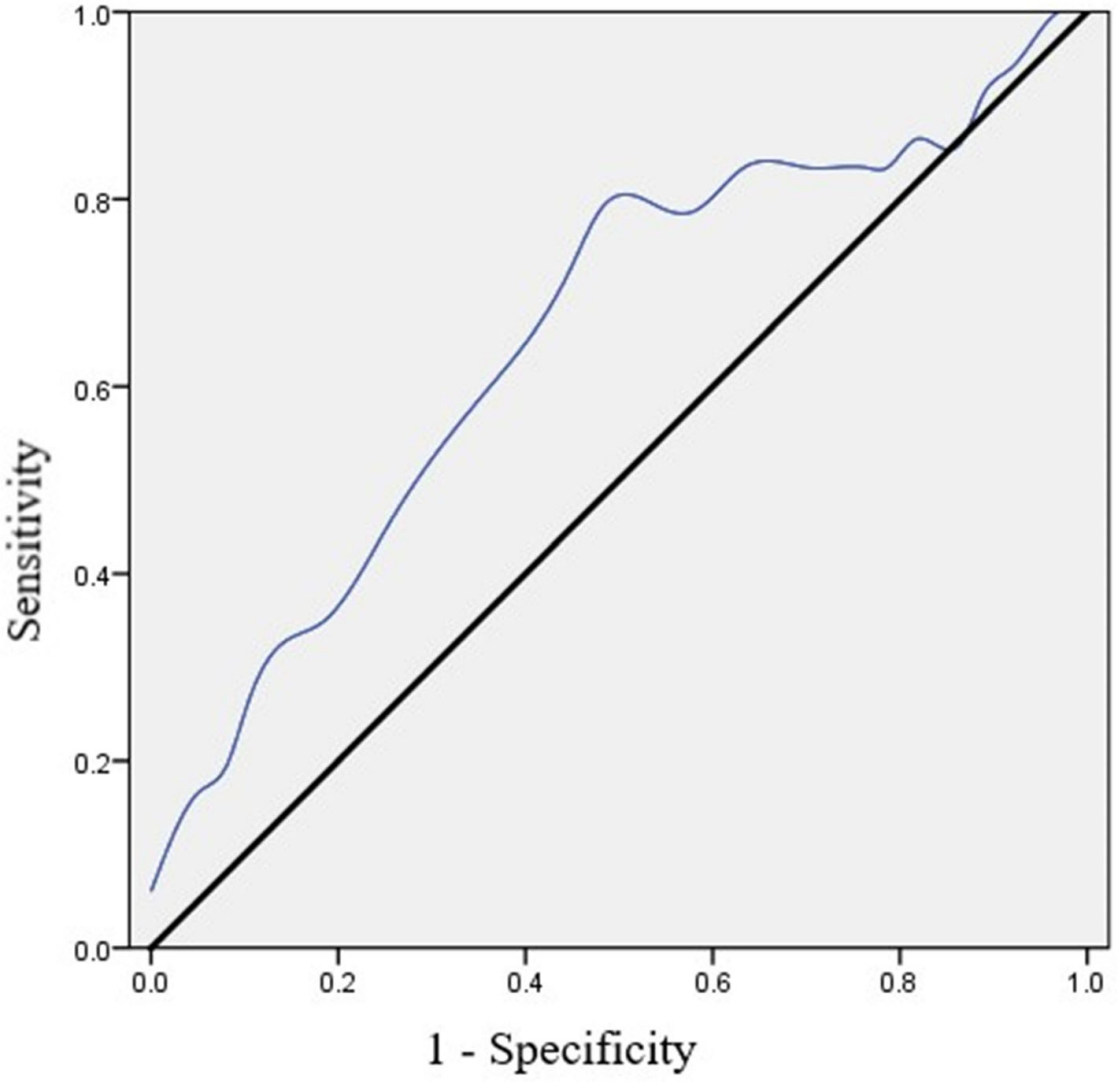
Receiver operating characteristic (ROC) curve of SHAPS in discriminating BD-II from MDD patients. Note: SHAPS = Snaith-Hamilton Pleasure Scale, BD = bipolar disorder, MDD = major depressive disorder

As shown in Fig. 1, we found SHAPS could differentiate BD-II from MDD patients. The sensitivity, specificity, FPR, FNR, and Youden index are shown in Table S1. According to the Youden index, our results indicated the best screening cutoff between BD-II and MDD was 26 (the value must be an integral number), with the AUC as 0.655 (*P* = 0.001, 95% CI = 0.568 to 0.742), and the corresponding SEN, SPE, FPR, FNR, and Youden index was 0.788, 0.520, 0.480, 0.212 and 0.308, respectively.

## Discussion

Discriminating BD from MDD is a major clinical challenge as misdiagnosis could directly affect the treatment and prognosis of patients. Thus, substantial studies have focused on finding biomarkers for early diagnosis of BD in depressive patients, and progress has indeed been made. However, those findings are far from clinical application. In clinical practice, symptom rating scales play important roles in assisting the diagnosis of diseases. Since evidence supports that MDD and BD patients displayed distinct characteristics of anhedonia, we aimed to compare the demographic and clinical differences between drug-free BD-II and MDD patients, and further to verify whether SHAPS, a tool used to evaluate anhedonia, could be used to help clinicians preliminary identify BD-II patients from MDD patients. The main findings of the present study were as follows: (1) Drug-free BD-II patients had more severe anhedonia, tend to be more extroverted, had a higher rate of drinking and smoking compared to drug-free MDD patients; (2) The stepwise logistic regression analysis revealed that the SHAPS score, drinking habit, and extroversion as influencing factors for the identification of BD-II; (3) SHAPS could differentiate BD-II from MDD patients with the AUC as 0.655, and the sensitivity was 0.788 at the best screening cutoff 26.

SHAPS is an excellent tool for assessing anhedonic symptoms in clinical and non-clinical populations [26–28], which is defined as ‘markedly diminished interest or pleasure in all, or almost all, activities most of the day, nearly every day [29]. Ample evidence supports that MDD and BD patients had more common and severe anhedonia compared to normal controls [30, 31]. Mazza et al. found that over half of patients diagnosed with BD experience significant levels of anhedonia during a depressive episode [32], and nearly 75% MDD patients also reported anhedonic symptoms [30, 33]. Those findings highlight the potential of reward processing deficits related to anhedonia to be trait factors of mood disorders. Interestingly, a recent study demonstrated that anhedonia was more frequent in BD patients compared to MDD patients [34], which is in line with our current findings but opposite to an earlier investigation reported that unipolar depressed patients exhibited greater severity of anhedonia [35]. However, most previous studies did not use specialized tools to assess anhedonia, and this might be the main explanation for the existing discrepancy.

Since anhedonia represents a deficit in reward processing, there have been many studies that reported abnormal reward processing in both MDD and BD patients. In addition, strong evidence indicated different reward processing dysfunction between MDD and BD patients. For instance, Manelis et al. found anticipation of loss was characterized by bottom-up fronto-striatal connectivity in MDD, and more sparse connectivity in BD that lacked fronto-striatal connections [36]. Redlich and colleagues observed that both BD and MDD patients had lower activity in the nucleus accumbens during reward processing, while BD showed a decreased activation, in the reward condition, of the nucleus accumbens, caudate nucleus, thalamus, putamen, insula, and prefrontal areas compared with MDD [37]. Furthermore, immune-inflammatory disturbances have been constantly implicated in the pathophysiology of reward-related disorders [38], and adjuvant anti-inflammatory therapy can significantly improve anhedonia in patients with unipolar and bipolar depression [39]. Interestingly, our previous work found that BD patients and MDD patients showed a distinct characteristic of immune inflammation [13], and immune system-related proteins may be used for distinguishing bipolar depression from MDD [14]. Moreover, as reward processing is modified under conditions of repeated stress, previous studies also suggest that alterations in glucocorticoid mechanism may contribute to anhedonia [40]. Indeed, prior evidence demonstrated the dysregulation of glucocorticoid system in mood disorder [41, 42], but also showed some differences between MDD and BD [43]. Taken together, these foregoing data imply that the anhedonia in MDD and BD may be distinct, and our current study further supported that BD-II at depressive episode had more severe anhedonia compared to MDD patients.

As we know, demographic characteristics did provide clinicians with complementary information for the diagnosis and differential diagnosis of depression to some extent. In the present study, we found that BD-II patients had a higher rate of alcohol use and smoking compared to MDD patients, which was consistent with previous research showed that smoking and alcohol use or dependence are more common in BD patients [44, 45]. Those results suggest that BD patients may suffer more substance abuse than MDD patients, and this adverse overall relationship between smoking, alcohol use, and BD, regardless of the direction of the effect, deserves major attention from clinicians. In addition, our results also showed that BD patients tend to be more extroverted than MDD patients, which was also reported early [46]. However, other studies failed to detect character differences between these two patient groups [47]. Hence, these relationships should be further explored in the future.

Some limitations in the present study should be considered. Firstly, owing to the relatively small sample size, especially the BD-II group, the conclusions that can be drawn from our data are limited. Secondly, all participants were recruited from outpatients, thus, our findings might not be generalizable to the inpatients. Thirdly, only a limited number of scales were used in our simple design study, it would be more effective when more symptoms rating scales were combined in identification of BD-II from MDD. Fourthly, the original MDD might be incorrect as the fact of the existence of the misdiagnoses of BD, so the cross-sectional design may lead to bias. Taken together, our preliminary finding should be interpreted with caution due to the above limitations, further study with a larger sample size will be necessary to verify our findings, and longitudinal design is also needed to track the clinical outcomes in patients with MDD.

## Conclusion

The present study provided evidence suggesting anhedonia difference between BD-II and MDD patients, and psychiatrists must pay more attention to anhedonic symptoms in depressive patients.

## Supplementary Information

**Additional file 1.**

## Data Availability

The datasets used and/or analysed during the current study are available from the corresponding author on reasonable request.
