# Supplementary material for: Anhedonia difference between major depressive disorder and bipolar disorder II
Source: BMC Psychiatry. 2021 Oct 27;21:531. doi: 10.1186/s12888-021-03548-w (PMC8555067; doi:10.1186/s12888-021-03548-w)
Supplement: Supplementary file 1 — Additional file 1. [file 12888_2021_3548_MOESM1_ESM.docx]

Table S1. Sensitivity, specificity, false positive rate, false negative rate and Youden index of SHAPS.

| Cutoff | 13.0 | 14.5 | 15.5 | 16.5 | 17.5 | 18.5 | 19.5 | 20.5 | 21.5 | 22.5 | 23.5 | 24.5 | 25.5 | 26.5 |
| --- | --- | --- | --- | --- | --- | --- | --- | --- | --- | --- | --- | --- | --- | --- |
| Sensitivity | 1.000 | 1.000 | 0.939 | 0.909 | 0.864 | 0.864 | 0.833 | 0.833 | 0.833 | 0.833 | 0.788 | 0.788 | 0.788 | 0.727 |
| Specificity | 0.000 | 0.031 | 0.082 | 0.112 | 0.133 | 0.184 | 0.214 | 0.235 | 0.296 | 0.367 | 0.418 | 0.449 | 0.520 | 0.551 |
| FPR | 1.000 | 0.969 | 0.918 | 0.888 | 0.867 | 0.816 | 0.786 | 0.765 | 0.704 | 0.633 | 0.582 | 0.551 | 0.480 | 0.449 |
| FNR | 0.000 | 0.000 | 0.061 | 0.091 | 0.136 | 0.136 | 0.167 | 0.167 | 0.167 | 0.167 | 0.212 | 0.212 | 0.212 | 0.273 |
| Youden | 0.000 | 0.031 | 0.021 | 0.021 | -0.004 | 0.047 | 0.048 | 0.068 | 0.129 | 0.201 | 0.206 | 0.237 | 0.308 | 0.278 |

| Cutoff | 27.5 | 28.5 | 29.5 | 30.5 | 31.5 | 32.5 | 33.5 | 34.5 | 36.5 | 38.5 | 39.5 | 40.5 | 41.5 | 43.0 |
| --- | --- | --- | --- | --- | --- | --- | --- | --- | --- | --- | --- | --- | --- | --- |
| Sensitivity | 0.606 | 0.455 | 0.348 | 0.318 | 0.258 | 0.197 | 0.167 | 0.121 | 0.091 | 0.076 | 0.061 | 0.045 | 0.030 | 0.000 |
| Specificity | 0.633 | 0.745 | 0.816 | 0.867 | 0.898 | 0.918 | 0.949 | 1.000 | 1.000 | 1.000 | 1.000 | 1.000 | 1.000 | 1.000 |
| FPR | 0.367 | 0.255 | 0.184 | 0.133 | 0.102 | 0.082 | 0.051 | 0.000 | 0.000 | 0.000 | 0.000 | 0.000 | 0.000 | 0.000 |
| FNR | 0.394 | 0.545 | 0.652 | 0.682 | 0.742 | 0.803 | 0.833 | 0.879 | 0.909 | 0.924 | 0.939 | 0.955 | 0.970 | 1.000 |
| Youden | 0.239 | 0.199 | 0.165 | 0.186 | 0.156 | 0.115 | 0.116 | 0.121 | 0.091 | 0.076 | 0.024 | 0.045 | 0.030 | 0.000 |

Note: SHAPS = Snaith-Hamilton Pleasure Scale, FPR=false positive rate, FNR=false negative rate.
